# Producer practices and attitudes: Non-replacement male calf management in the Australian dairy industry

**DOI:** 10.3389/fvets.2022.979035

**Published:** 2022-09-20

**Authors:** Veronika Vicic, Anthony J. Saliba, Michael A. Campbell, Gang Xie, Jane C. Quinn

**Affiliations:** ^1^School of Agricultural, Environmental and Veterinary Sciences, Charles Sturt University, Wagga Wagga, NSW, Australia; ^2^Gulbali Institute for Agriculture, Water and the Environment, Charles Sturt University, Wagga Wagga, NSW, Australia; ^3^School of Psychology, Charles Sturt University, Wagga Wagga, NSW, Australia; ^4^Quantitative Consulting Unit, Charles Sturt University, Wagga Wagga, NSW, Australia

**Keywords:** producer wellbeing, dairy producers, euthanasia, Bayesian network, producer attitudes, non-replacement male calf

## Abstract

Currently, there is no standardized rearing method or production guidelines for non-replacement male dairy calves that maximizes their economic viability. Producers have highlighted the need to match consumer expectations, but even with broadscale welfare improvement across the dairy industry, challenges remain at providing reliable and valuable pathways for non-replacement male dairy calves for beef production. A key consumer concern has been the use of on-farm euthanasia. Euthanasia has been a catalyst for change in the industry from a human and animal welfare perspective. The practice of euthanasia can lead to a decline in personnel wellbeing. To investigate the relationship between on-farm management practices of non-replacement male dairy calves and producer perceptions of their value proposition, an online questionnaire was provided to Australian dairy producers between June and October 2021. The aim was to identify supply-chain profitability of non-replacement male calves and investigate the attitudes and effects of euthanasia on producer wellbeing as part of managing these calves. A total of 127 useable responses were obtained, and a Bayesian network (BN) was utilized to model the interdependencies between management practices and wellbeing among participants. The results indicated that in general, dairy producers desired high welfare standards in their enterprises with regard to non-replacement male calves as well as expressed a desire to meet industry and consumers' expectations. In line with anecdotal reports of a reduction in practice, euthanasia was not identified as common practice in this group; however, producers were still accessing early-life markets for non-replacement male calves with operational requirements and environmental factors influencing their decisions. Producers expressed dissatisfaction with market access for their calves, as well as the lack of suitability of Australian beef grading standards for dairy-bred carcasses. Australian dairy managers and owners identified that euthanasia influenced employee wellbeing; however, they did not acknowledge euthanasia had an effect on their own wellbeing. Overall, the findings of this study indicate that all non-replacement male calf breeds had the potential to access profitable markets, and avoidance of euthanasia is a strong driver of change among dairy beef production systems in Australia.

## Introduction

Current dairy production generates non-replacement calves as part of its standard operating practices (1). Despite the predictable and continuous production of non-replacement male calves, there is currently no standardized rearing method or production guidelines for maximization of their economic potential. Due to competing economic demands, mixed practices currently exist in the management of non-replacement male dairy calves, including that healthy non-replacement male calves may be euthanized at a young age, commonly <10 days of age (2, 3). At the same time, consumers are becoming more aware of the systems that produce the food and dairy products they consume (4). Consumer sentiment is driving practice change globally. There is preference for non-replacement calves to be utilized in the beef supply chain as opposed to being destined for early-life slaughter (5).

Non-replacement male calves (colloquially known as “bobby calves” in Australia) are generally male dairy-bred calves <30 days old or weighing <80 kg (6). Currently, a number of pathways exist for these bobby calves, including on-farm rearing and rearing by intensive calf producers, to generate a “dairy beef” product, and it is estimated there are approximately 400,000 bobby calves processed each year in Australian abattoirs (6). Despite advances, these production pathways are often disaggregated in relation to intensive labor requirements, on-farm calf rearing facilities, and low-value saleable markets. This is also seen in dairy production systems in America and Canada, where similar issues have led to poor calf welfare and lack of adoption of on-farm rearing (7, 8). A shift has occurred in societal attitudes towards the practices surrounding non-replacement male calves and the need to identify alternative strategies to rear, process, and market a viable and economically sustainable dairy beef product. This has led to increased interest in providing valuable pathways for bobby calves nationally and internationally (9) with industry and the consumer driving practice change to achieve alternative outcomes for non-replacement male calves (10). There have been attempts to avoid the need for euthanasia through the use of sexed semen, although this has not proven to be a complete solution due to lower conception rates and higher production cost (11). Another opportunity to avoid euthanasia of bobby calves is to market the potential of their favorable eating quality attributes; however, Jersey calves are still perceived to have poor muscling and growth, thus impacting economic viability (3).

In Australia, dairy producers have highlighted their intention to continually improve production practices for rearing of non-replacement male calves; however, our previous work and that of others have identified that the lack of economically sustainable dairy beef pathways can still lead to unavoidable euthanasia (8, 12). Similarly, internationally, the realities of overcoming supply-chain issues are complex (13), and no industry-wide solutions exist for producers to have reliable pathways to market for these animals (12), leaving euthanasia uncommon, but still practiced. In 2022, it was reported that the number of non-replacement calves processed in Australian abattoirs has reduced from approximately 450,000 calves per year to 300,000 calves. However, this has been influenced by the increase in beef cattle prices at the time, and the number may not continue to reduce if cattle prices were to decrease again in future (14).

Euthanasia of economically non-viable livestock represents an ethical challenge from many perspectives. Personnel directly involved in performing euthanasia of livestock have been reported to show conflicting views toward the practice, desiring to achieve a positive economic and/or welfare outcome, while accommodating an undesirable need to perform euthanasia as part of a management system (15). This dichotomy can lead to “moral stress” with significant impacts on those staff involved (15, 16). While it has been identified by producers that in some cases, euthanasia is morally right, for example, where retention of these animals may lead to poorer welfare outcomes, these practices still create a moral tension for those involved. The impacts of these practices in the dairy industry are still largely unknown (8, 17). Secondary trauma caused by repeated decisions to euthanize healthy animals has been associated with burnout, moral injury, and, in some cases, post-traumatic stress disorder (3, 18).

A questionnaire was developed to investigate Australian dairy producers' practices and attitudes surrounding non-replacement male calf management inclusive of past, present, and future euthanasia practices and examine the perceived impacts on their wellbeing. This study also aimed to identify producer practices and potential markets that could maximize profitability for non-replacement male calves within the dairy beef supply chain as well as investigating producer opinions surrounding the market acceptability of beef products. To integrate the relationships between the composite sections of the questionnaire, a Bayesian network (BN) model was established and inferential analysis was conducted using Netica (19). This allowed determination of relationships between the demographic and operational responses from participants of the questionnaire and their impact and influence on wellbeing parameters.

## Materials and methods

Ethical approval for the collection of original data from human participants *via* an online questionnaire was provided by the Charles Sturt University Human Research Ethics Committee (Protocol number: H20352). This work was carried out in full compliance with the National Statement on Ethical Conduct in Human Research (2007, updated 2018) and in accordance with the National Health and Medical Research Council Act (1992). All participants implied consent to participate in the questionnaire by proceeding to respond to the questionnaire after receiving a consent statement.

### Questionnaire design, recruitment, and participants

The questionnaire design was built on previous qualitative findings described by Vicic et al. (8) who identified attitudes and practices surrounding on-farm non-replacement male calf production, euthanasia practices, and producer wellbeing. Through mixed methods research strategies, the outcomes from the qualitative interviews informed the directive of each item developed for the online questionnaire presented in this study (20, 21). The study was also aligned with the requirements for reporting observational studies using the Strengthening the Reporting of Observational Studies in Epidemiology (STROBE) guidelines (22). The items in the questionnaire had face validity. An iterative process was carried out that consisted of piloting and revising each version of the questionnaire by the research team, which included a perceptual psychologist and production animal scientists. The final draft of the online questionnaire was piloted with a dairy veterinarian and previous and current dairy owners. This process ensured relevance of the questionnaire to the research aims and meaningfulness to potential participants.

The online questionnaire was administered *via* SurveyMonkey^TM^ (http://www.surveymonkey.com) and managed by the Spatial Data Analysis Network (SPAN) at Charles Sturt University. The questionnaire was distributed *via* social media and through industry organizations between June and October 2021. Purposive criterion sampling was used to recruit Australian dairy owners and/or managers older than 18 years. Participants who did not meet the criteria were excluded *via* screening questions at the start of the questionnaire. Participation in the questionnaire was by implied consent, voluntary, and anonymous. Specifically, an information sheet was provided prior to the start of the questionnaire, and consent was implied if the participant chose to proceed. A $20 gift card was offered to each participant as a token of appreciation for engaging in the study if contact details were provided for this purpose. Participant contact details were held separately to the participant's questionnaire results to ensure data anonymity.

### Measures

The questionnaire (see the Supplementary material) was structured to include the following categorized items:

#### Demographics

The demographic items in the questionnaire included age, gender, postcode, and education level. Items measuring the participants' involvement in the dairy industry and enterprise include location, milking herd size, and breed.

#### Practices and market orientation

The participants were asked questions relating to their past, present, and future practices relating to non-replacement male calves and market accessibility for specific breed types and their associated profitability. Perceptions surrounding perceived quality and marketability of dairy beef products were also measured.

#### Attitudes and perceptions

The attitude component of the questionnaire asked a range of questions that explored the impact of undertaking of euthanasia on the participant wellbeing and mental health, satisfaction of on-farm practices, and supply-chain access for non-replacement male calves.

#### Personal and psychological wellbeing

The Personal wellbeing index-Adult (PWI-A) (23) was used as one of two validated measures to assess dairy producer wellbeing. The PWI-A scale focuses on the quality of life of the individuals. The Depression Anxiety and Stress Scale (DASS-21) (24)—short version, was the second validated tool used for measuring depression, anxiety, and stress among the participants. The DASS-21 is a reliable and widely used clinical screening tool for these constructs (25).

#### Statistical analysis

Data from the online questionnaire were exported into Microsoft Excel (26). Only completed entries were considered for analysis. Remaining entries were cleaned of duplicates, partial responses (91 responses), and ghost submissions (87 responses), as well as checked for errors. To assist in data analysis, open-ended responses were assigned one or more words from a code list to allow for categorization of responses. The code list was created by coding the raw short answer responses to engage in data reduction and simplification. A list of codes was then developed with associated definition and assigned to the short answer responses (27). Descriptive statistical analyses were conducted in R (28) to summarize and better understand the demographics, practices, and attitudes as well as wellbeing of participants.

#### Bayesian network model

Bayesian network (BN) models are an intuitive, graphical representation of a joint probability distribution of a set of random variables that are used to capture possible mutual causal relationships, where each node represents a variable and the directed link edges denote the dependency relationship between those variables (29, 30). The Bayes theorem underpins BNs and can be defined by the following mathematical formula:


$$\begin{array}{l} \mathrm{Pr}\;\left(\text{B }|\text{ A}\right)=\frac{\mathrm{Pr}\;\left(\text{A }|\text{ B}\right)\mathrm{Pr}\left(\text{B}\right)}{\mathrm{Pr}\left(\text{A}\right)}=\;\frac{\mathrm{Pr}\left(\text{A},\text{ B}\right)}{\mathrm{Pr}\left(\text{A}\right)}, \end{array}$$

where A and B are two random variables; Pr(A) and Pr(B) are the marginal probability distributions of A and B, respectively; Pr(B| A) is the conditional probability distribution of B given A; Pr(A| B) is the conditional probability of A given B; and Pr(A, B) is the joint probability distribution for A and B (31). A BN model using Netica software (19) was developed to provide an mathematically coherent framework for the analyzing the complex associations between producer responses to practices and attitudes reported in the questionnaire. BNs allow a level within multiple nodes to be selected as the target variable(s). Given the selected level of a target variable, the expected probability distributions of other variables can be assessed. For example, by fixing the values of some variables (equivalent to those predictor variables in a regression model), we can estimate/predict the values (or the distribution of the values) of the remaining variable(s) in the BN model (32).

**Figure 1:**
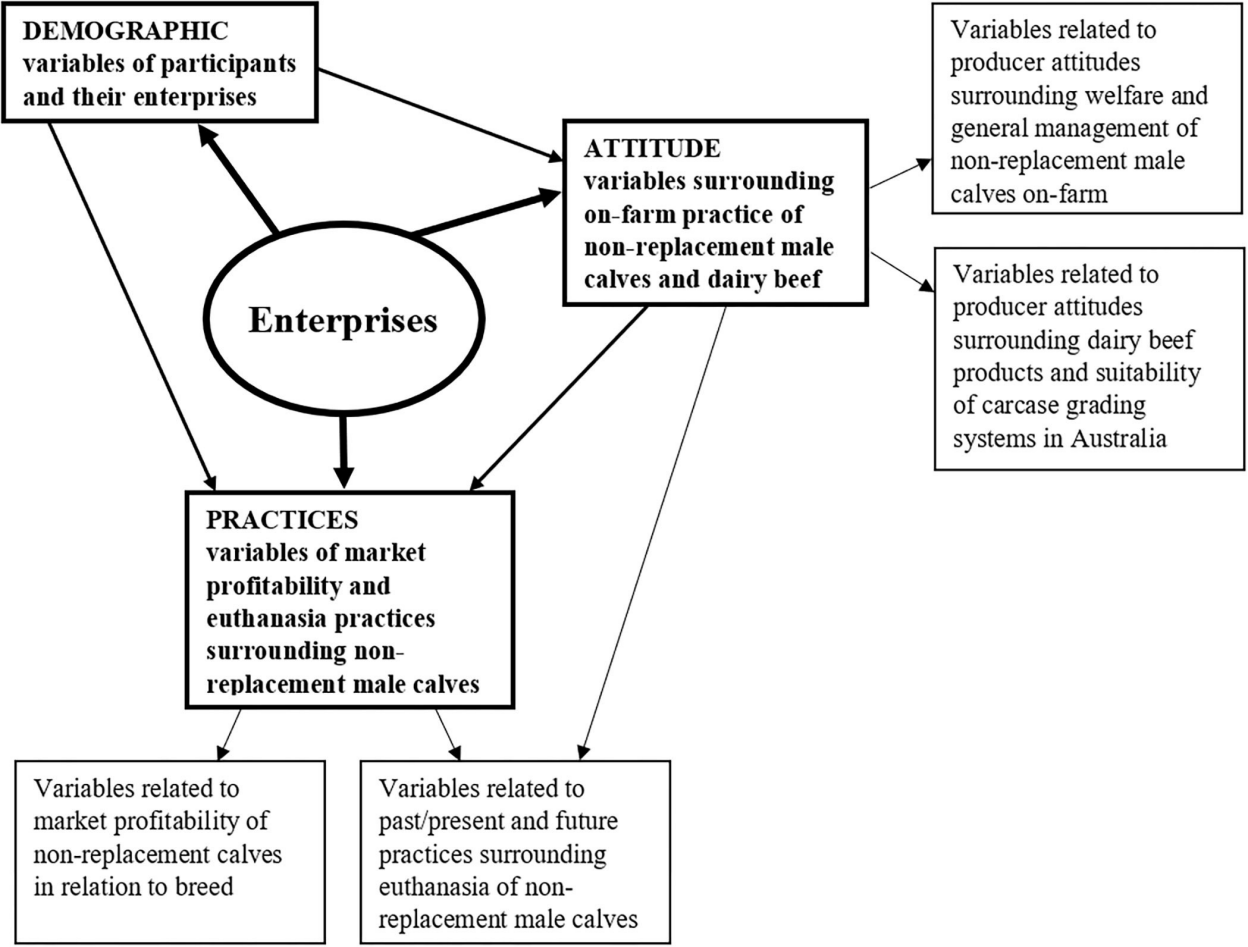
Schematic diagram of the conceptual model that informed the development of the Bayesian network to describe the interrelationships between producer demographics, practices, and attitudes of non-replacement male calf management in the Australian dairy industry.

The development of a BN model started from building a conceptual model (Figure 1) that identified the interdependent relationships of those variables of our research interest upon the collected data and the researcher's disciplinary knowledge.

**Figure 2:**
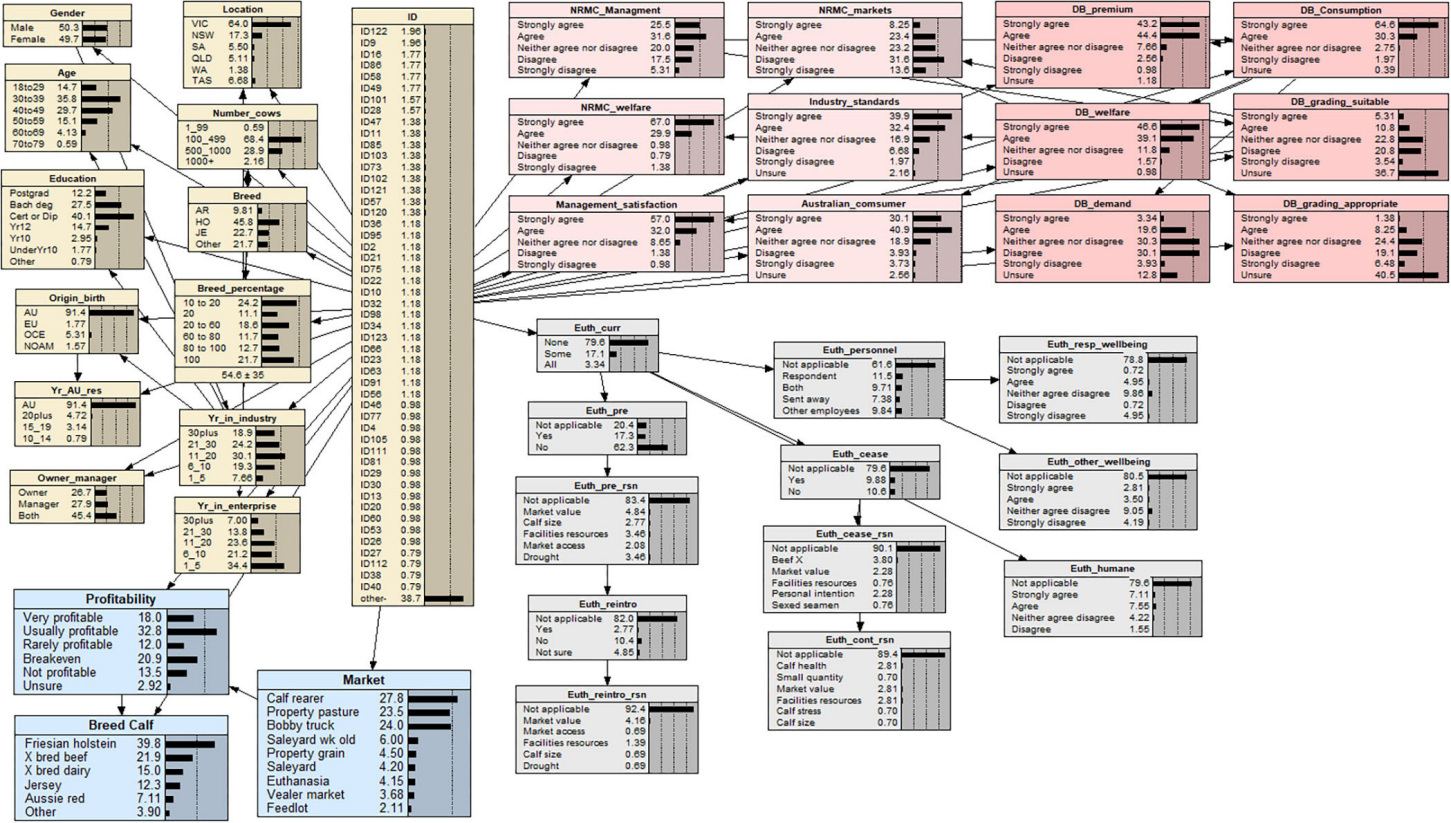
A Bayesian network (BN) model of the dependency relationships between practice and attitude variables of Australian dairy producers (127 participants) regarding non-replacement male calves. Each variable in the BN model is represented by a node. The link between two nodes represents the dependency relationship between two variables. The middle column of each node is a percentage totaling to 100%, which represents the analysis outcomes of each level within a node. The last column is a graphical representation of the percentage values for each level shown as distribution bars. The dotted lines are markers that are equally spaced to aid in visualizing the comparative heights of the distribution bars. Descriptions of each variable and their levels are presented in Supplementary Table 1.

Every BN model has two components in its model specification. The qualitative component of a BN specifies the network structure by connecting all the variables/nodes in the model; the quantitative component of a BN determines the conditional probability tables (*via*., evaluating the parameters of a BN model), which quantifies the strengths of dependence relations using the probability theory (19, 29, 30). In this study, the BN model was developed through a multi-step hybrid procedure. In the model structure specification step, a hybrid approach was adopted. Based on the conceptual model as schematically shown in Figure 1, four sub-models were specified, among which three were determined using the Netica built-in machine learning algorithm (the tree augmented naive Bayes network algorithm) (19), and the fourth one was manually built according to our disciplinary knowledge. With “enterprises” as the target variable, the three machine learning sub-models were (1) sub-model of 13 demographic variables; (2) sub-model of 12 attitude variables; (3) sub-model of three variables of market profitability; and the fourth sub-model related to the 12 practices surrounding euthanasia was built manually. To complete the model structure specification step, the net merge function in Netica was applied to combine the four sub-models into one coherent BN model. Finally, the expectation–maximization (EM) algorithm in Netica was employed to complete the model parameter estimation step. EM learning takes a BN and repeatedly uses the model to find a model of best fit using expectation (E) and maximization (M) steps. Expectation uses regular Bayes network inference with the existing Bayes network to compute the expected value of all the missing data. Maximization finds the maximum likelihood in the BN using the original data plus expected value of missing data (32). The resulting BN model is presented in Figure 2.

The BN model (Figure 2) was a statistical representation of the interdependencies between producer demographics, practices, and attitudes surrounding non-replacement male calves as well as market profitability of non-replacement male calves. The model contained 40 observed variables/nodes: 13 demographic-related variables (light yellow color nodes), 12 euthanasia-related variables (gray color nodes), 12 attitude-related variables (pink nodes: six regarding animal management and welfare and six regarding dairy beef), and three blue color nodes relating to markets of non-replacement male calves and associated profitability (Figure 2). Skip logic in the euthanasia section of the questionnaire resulted in specific questions not being required to be answered, as such “not applicable” was assigned to participants who did not respond in these instances.

Sensitivity to findings is a built-in function in Netica that enables selection of a target variable and ranks the level of influence of all other variables (32). This function was used to rank the strength of the associations between variables from highest to lowest (19). Several competitive BN models including latent variables (i.e., demographics, practice, attitude, and market) were compared against the original model. However, after performing sensitivity to findings test, all the latent variables resulted in non-meaningful categorization; hence, the final BN model did not assume latent variables. Demographics were found to be heavily influenced by gender, so female and male levels were selected to determine the demographic difference associated with each gender. The observed variable for practice was selected to be “Euth_curr”; however, the levels “some” and “all” were designated to be evenly weighted in the BN model for enterprises that currently practiced on-farm euthanasia of some or all non-replacement male calves. This was due to the small proportion of participants who currently euthanized on farm. The selected model structure was chosen as it was deemed to be the most optimal way to summarize the data in a contextual meaningful way that allowed logical inference of relationships. Based on the Bayes theorem or the law of conditional probability, the BN model (Figure 2) characterized local dependence relationships between two nodes/variables and then linked all variables as a network according to the chain rule of probabilities, hence providing a holistic representation of questionnaire data that show defined statistical associations (19, 33).

### Demographics

The questionnaire received a total of 127 completed online responses; however, due to the distribution through third-party organizations, the response rate could not be calculated. Questionnaire responses were received from each state in Australia; Victoria = 64%, New South Wales = 17% Queensland = 6%, South Australia = 6%, Tasmania = 5%, and Western Australia = 3%. The proportion of responses by state was similar to the proportion of dairy enterprises in Australia by state, suggesting appropriate regional representation (34); our sample of 127 represented 2.2% of dairy farms in Australia.

Based on the outcomes of the BN model, both genders had an even distribution of owners and managers and were predominately located in Victoria. The male participants of the questionnaire were likely to be middle-aged and have worked a greater number of years in the dairy industry but lesser years in the current enterprises they were owning and/or managing. The male participants were also likely to have a lower level of education than female participants. The female participants were likely to be older in age than the male counterparts and owned and/or managed a dairy herd with a higher number of milking cows, with the predominate breed in each herd being Holstein. Probabilities for demographic response variables are summarized in Table 1 when gender (male and female) was selected as the target variable in the BN model.

**Table 1:** Percentages reported for demographic responses of Australian dairy producers in the Bayesian network model when different levels for gender (male and female) were selected as the target variables.

		**Gender**	
**Category**	**Levels**	**Male (%)**	**Female (%)**
Owner/Manger	Owner Manager Both	25.4 28.5 46.1	28.1 27.3 44.7
Age	18–29 30–39 40–49 50–59 60–69 70–79	13.7 35.5 35.9 12.1 1.56 1.17	15.8 36.0 23.3 18.2 6.72 0+
Location of enterprise	NSW VIC QLD TAS SA WA	19.1 54.7 10.2 9.77 6.25 0+	15.4 73.5 0+ 3.56 4.74 2.77
Years in dairy industry	1–5 6–10 11–20 21–30 30 plus	5.47 11.7 37.5 30.5 14.8	9.88 26.9 22.5 17.8 22.9
Year in current enterprise	1–5 6–10 11–20 21–30 30 plus	41.4 18.0 28.9 4.92 6.80	27.3 24.5 18.2 22.8 7.20
Education level	Under year 10 Year 10 Year 12 Certificate or Diploma Bachelor's degree Postgraduate degree	3.52 2.74 11.7 49.6 22.7 9.77	0+ 3.16 17.8 30.4 32.4 14.6
Number of cows in milking herd	1–99 100–499 500–1000 1000 plus	.001 74.2 23.8 1.95	1.19 62.4 34.0 2.37
Breed of milking cows	Holstein Jersey Aussie Red Other	48.1 23.8 8.69 19.4	43.5 21.6 10.9 23.9

### Practices regarding non-replacement male calves

Practices regarding non-replacement male calf management were divided into three descriptive categories: (1) enterprises that did not euthanize non-replacement male calves on farm, (2) enterprises that euthanized some non-replacement male caves on farm, and (3) enterprises that euthanized all non-replacement male calves on farm. Most participants (84%; 107/127) indicated that they did not euthanize non-replacement male calves on farm; 13% (16/127) of participants indicated that they sometimes euthanized calves; and only 3% (4/127) indicated they euthanized all non-replacement male calves in their enterprise.

The BN model indicated the cohort of participants who were managing and/or owning enterprises that previously euthanized on farm (21.7%) selected the response that indicated they were likely to do so because of the low market value of calves, lack of facilities and resources to rear calves, and the impacts of drought. A majority of these participants indicated their enterprises would not reintroduce euthanasia (55%), but some participants from this cohort were unsure (24%) or would (16%) reintroduce euthanasia if the market value of calves decreases, and they were unable to support production such as during drought conditions.

From the cohort of participants who currently euthanize some (17.1%) or all (3.34%) non-replacement male calves on farm, the majority (64.4%) was not likely to cease euthanasia on their enterprises for similar reasons as mentioned previously. The participants who indicated they were in the process of ceasing euthanasia as a practice were doing so due to the increase in beef over dairy calves in their herd, the increase in the market value of calves, and their own personal decision to cease euthanasia. Commonly, both owners/managers and employees of each enterprise were the personnel involved in performing on-farm euthanasia. The participants were likely to strongly agree (40.9%) or agree (28.8%) that their euthanasia practices were humane but also had clear intentions to refrain from euthanasia, where possible.

### Producer wellbeing

The relationships between personnel practicing euthanasia and the effects of practicing euthanasia on wellbeing and mental health were assessed in the BN (Figure 2, gray nodes). A total of 20 participants reported that euthanasia was still practiced on farm. From this cohort of participants, 25% claim that practicing euthanasia does not influence their own wellbeing and mental health. Of the 20 participants, 19 indicated they allocated the role of euthanasia to staff as well, but one participant indicated they were the only person on their enterprise who carried out euthanasia. Similarly, 31.4% of the participants indicated that euthanasia does not influence their employees' wellbeing and mental health; however, a large proportion (19.2%) of these participants also stated that euthanasia does affect employee wellbeing and mental health. Similar findings were reported by the participants whose enterprises previously euthanized non-replacement male calves on farm.

**Figure 3:**
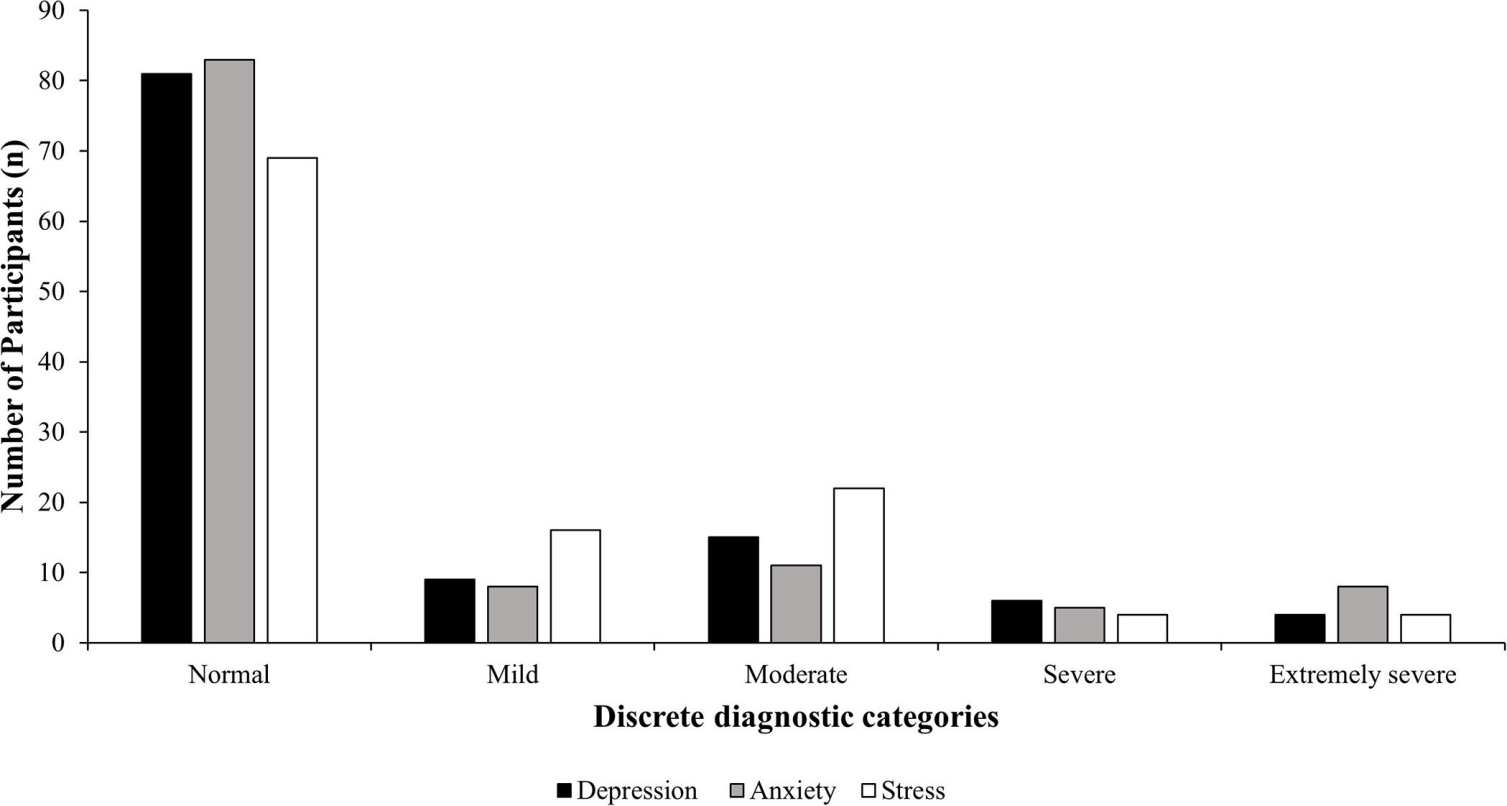
Participants' DASS-21 discrete diagnostic category results in relation to depression (solid black), anxiety (solid gray), and stress (solid white).

In addition to this, two validated psychological wellbeing tools were used to assess the psychological status of the participants. Of the 115 participants who responded to questions structured within the DASS-21, most participants fell within the “normal” range for depression (70%; 81/115), anxiety (72%; 83/115), and stress (60%; 69/115) on the DASS-21 scale (Figure 3, Table 2). Minor differences were found for severity of depression, anxiety, and stress in each DASS discrete diagnostic category among outcomes for the female and male participants.

**Table 2:** Scale reliabilities of DASS-21 and PWI-A.

**Characteristic**	**Current study**		**General population norms** ^**a**^		**Normative ranges** ^**b**^	
**Scale**	**M**	**SD**	**M**	**SD**	**(-2SD)**	**(+2SD)**
DASS-21 Depression DASS-21 Anxiety DASS-21 Stress	7.03 5.25 13.25	8.07 6.90 8.63	2.57 1.74 3.99	3.86 2.78 4.24		
PWI	73.93	15.9	75.5	13.9	74.2	76.8

DASS, Depression Anxiety Stress Scale; PWI-A, Personal Wellbeing Index-Adult. ^a^Australian general population norms taken from Crawford et al. (25); Capic et al. (40). ^b^Lower bound (-2SD) and the upper bound (+2SD) of the normative ranges were derived from the overall data, including surveys 3-34 [Capic et al. (40)].

However, in comparison to previous studies using Australian general public population normative values (Table 2) (25), the mean scores for each category within the DASS-21 were marginally higher among this cohort of Australian dairy producers than would be expected within the general population, potentially indicative of some psychological impact of their profession. Specifically, mean DASS-21 scores for all participants were at the high end of the “normal” category cutoff (Table 2), placing dairy producers at risk for depression, anxiety, and stress. Similarly, when the 115 participants undertook the validated Personal Wellbeing Index (PWI-A) psychometric instrument, the mean PWI-A score in this cohort of participants was 73.93, marginally lower than the general population mean of 75.5. These results indicated that personal wellbeing of this cohort does not lie within the respective normative range (74.2 to 76.8); therefore, these participants may be experiencing a lower quality of personal wellbeing than the general population (Table 2).

### Calf management and welfare attitudes

Questionnaire responses indicated (Figure 2, pink nodes) that the participants were likely to strongly agree or agree (96.9%) that welfare is of high importance when managing non-replacement male calves and that the participants were likely (89%) to gain satisfaction when they have good management practices in place. The participants also strongly agreed or agreed that they want to satisfy industry standards (72.3%) when managing non-replacement male calves as well as satisfy Australian consumers (71%). The participants were likely to strongly agree or agree (57.1%) that they are satisfied with their non-replacement male calf practices. However, the cohort of participants who euthanize some or all non-replacement male calves are likely to have selected neither agree nor disagree (45%) that they are satisfied with their management practices.

### Markets and profitability

**Figure 4:**
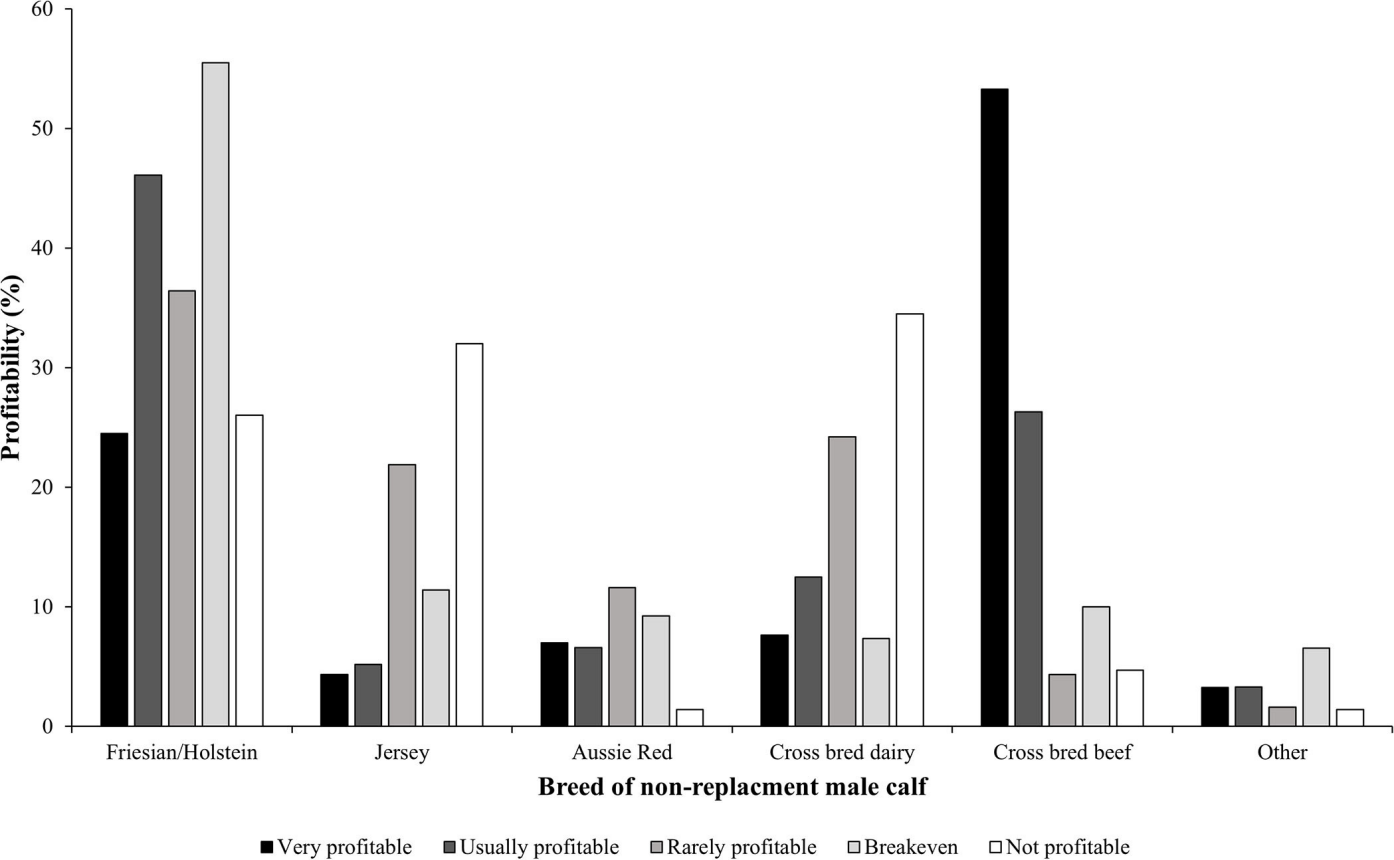
Profitability of breeds of non-replacement male calves reported by Australian dairy producers in 2021 (probabilities reported from the Bayesian model). The color gradient is represented by the following profitability categories from darkest to lightest; very profitable, usually profitable, rarely profitable, breakeven, and not profitable.

**Figure 5:**
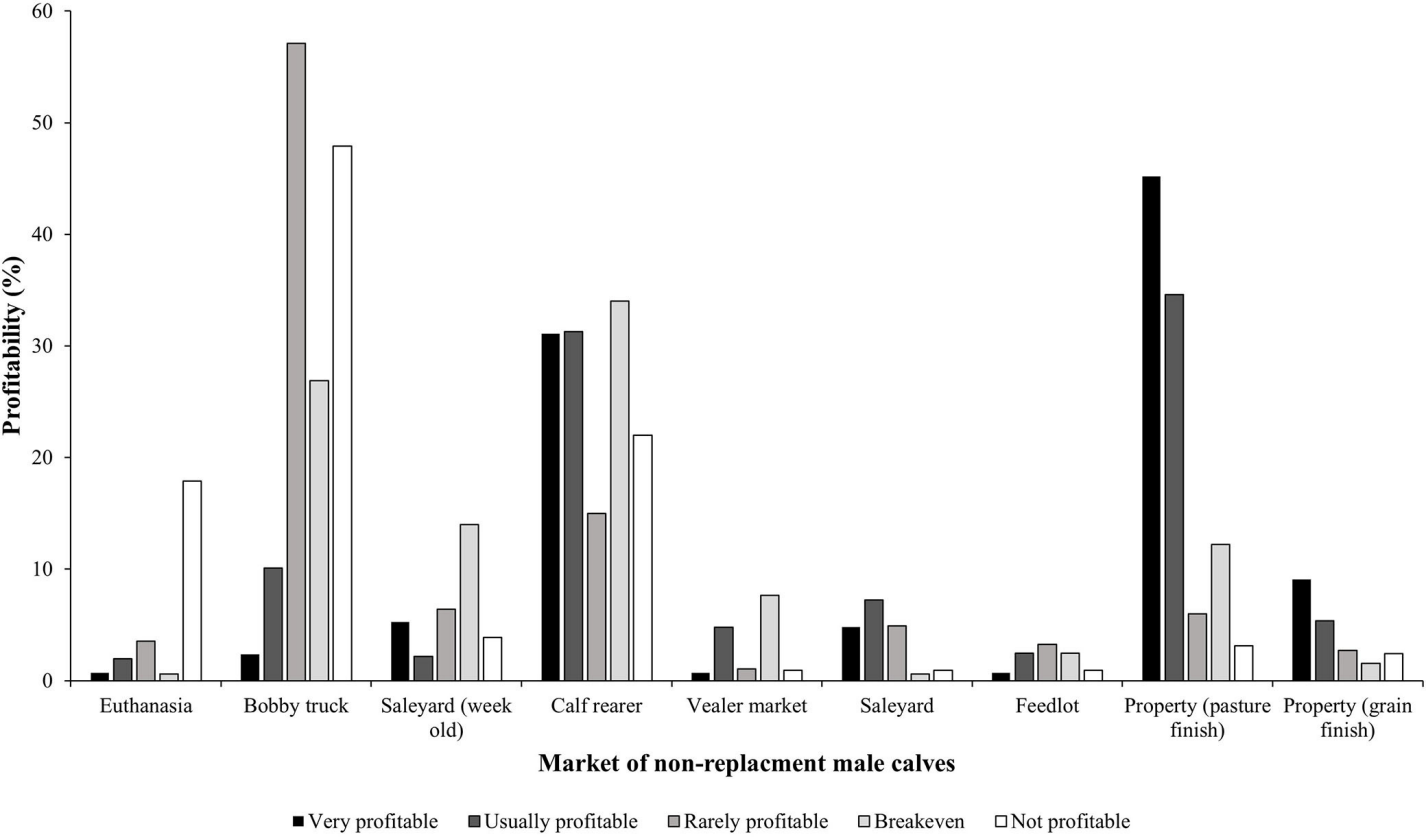
Profitability of the market accessed for non-replacement male calves reported by Australian dairy producers in 2021 (probabilities reported from the Bayesian model). The color gradient is represented by the following profitability categories from darkest to lightest; very profitable, usually profitable, rarely profitable, breakeven, and not profitable.

A majority of the participants were not satisfied with the markets they can access for their non-replacement male calves. The BN indicted that the most profitable combination for non-replacement male calves was likely to be a beef cross dairy calf (53.3%) or of Friesian/Holstein origin (24.5%) (Figure 4); finished at the property of origin on pasture (45.2%); or sold to the calf rearers (31.1%) (Figure 5). This finding suggests that growing calves out to steers can be viable if there is the local availability of land to do so. Interestingly, some participants reported euthanasia to be profitable (2%). This could be due to their perception that rearing non-replacement male calves would lead to further expenses, and therefore, euthanasia would be perceived as more profitable. The least viable combination for non-replacement male calves was likely to be cross bred dairy calves (34.5%) or of Jersey origin (32%) (Figure 4); those sent on a bobby truck (47.9%); sold to a calf rearers (22.0%); or euthanized on farm (17.9%) (Figure 5).

### Producer attitudes to dairy beef

Dairy beef, as a consumer product, is not widely available or marketed in Australia; therefore, producer perceptions were assessed regarding the potential value of dairy beef products by Australian consumers. The BN model indicated that the participants believed dairy beef products could both target a premium beef market (87.6%) and be marketed as a welfare-friendly product (85.7%) (Figure 2, pink nodes). A majority of the participants agreed they would consume beef from a dairy-origin animal (94.9%); however, they were likely to disagree that there was a consumer demand for dairy-origin beef products (34%).

The questionnaire presented several questions to determine if dairy producers considered that non-replacement dairy calves were suited to Australian carcass grading standards. A majority of the participants were unsure of the use of Australian carcass grading standards (36.7%), but the remaining participants agreed with the response that this grading system was not suited to dairy-origin carcasses (24.3%). Specifically, 25.5% of the dairy producers indicated that abattoirs did not grade carcasses from dairy-bred animals appropriately.

## Discussion

This quantitative study used a Bayesian network (BN) model as a mathematically coherent framework to explore the complex interrelationships (35) of Australian dairy producers' practices and attitudes surrounding non-replacement male calf management inclusive of euthanasia practices and examined the perceived impacts on producer wellbeing. The network can express association and interactive changes among variables to facilitate understanding the relationships between all variables in the model. As the questionnaire data represented a relatively small sample size (*n* = 127), the BN network advantageously shows in-sample prediction accuracy of results through EM learning (36). Our study had a relatively small sample size, which can introduce potential bias. The sample is representative of 2.2% of all dairy enterprises in Australia; however, the distribution of responses across each state was in proportion to dairy enterprises in each region (34). The study also could not achieve complete randomization in the sample obtained, also leading to potential selection bias (37).

Euthanasia of non-replacement calves was not identified as an industry-wide practice in this study. Only 16% of participants identified euthanasia as a current practice. The main reason for euthanasia was due to the lack of economically profitable markets to access for non-replacement male calves, a lack of rearing facilities and recourses, and/or impacts of drought on their system. The participants indicated the desire to eliminate euthanasia, where possible. Feelings associated with euthanasia of non-replacement male calves has been linked to a range of negative connotations where words such as “avoid, frustrated, hate, not supportive, refuse, unethical, and unpleasant” are often expressed when considering this topic (8). Anger, grief, and depression are emotions also known to be associated with euthanasia of dairy cattle, which may, if persistent, lead to compassion fatigue (3, 38). A few participants in this questionnaire indicated a preference to perform euthanasia of non-replacement male calves with the intent to eliminate poor welfare practices surrounding animal management, despite evidence of poor emotional outcomes associated with this practice (15, 17).

A majority of producers were satisfied with the management of non-replacement male calves; however, the cohort of participants who still practice euthanasia were likely to have selected neither agree nor disagree regarding satisfaction of management practices surrounding calves. The participants acknowledged negative mental health and wellbeing effects on employees who practice euthanasia on farm, although the negative effects of euthanasia did not influence the participants themselves. While not identified as a negative influence on themselves in the short term, long-term euthanasia practice could have negative effects on individuals (16, 38); such effects might be magnified as social attitudes change toward greater transparency and ethical practice in food production. The producers have acknowledged that formal training in euthanasia practices can reduce the impacts of negative wellbeing among personnel involved in the practice (8). Formal training can provide knowledge on correct, effective, and humane strategies to euthanize calves and, in turn, assist employees to be confident in this practice and decrease compromised emotional and wellbeing factors (17). In this context, euthanasia as a cause of lower wellbeing cannot be out ruled; therefore, further research is needed to determine whether euthanasia might cause long-term negative effects on those who practice or oversee it. Tailored service delivery programs targeting mental health in rural communities may be required, where high leaves of psychological health are compromised (39).

General wellbeing and psychological attributes were assessed among the participants of this study. The DASS-21 and PWI results placed a majority of dairy producers in the normal category for depression (0–9), anxiety (0–7), and stress (0–4); however, the overall means reported in this study for each diagnostic category (Depression 7.03, Anxiety 5.25, Stress 13.25) were higher than the mean scores reported for the Australian general population (Depression 2.57, Anxiety 1.74, Stress 3.99) (25). In addition to this, the mean wellbeing score (73.93) reported for this study was outside the normal range (74.2–76.8) of the general Australian population (40). There is a difference presented in wellbeing parameters among dairy producers and the Australian general population; however, this could be attributed to rural living, rather than work-related experiences. Poorer mental wellbeing has previously been linked to living in rural locations (41), and poorer health outcomes in general have long been associated with rurality in Australia (42). In particular, farming populations have also been identified to experience poorer mental health due to lack of access and distance to mental health services as well as stigma surrounding poor mental health more generally (39, 43). In this study, 7–11.5% of the participants fell in the severe and extremely severe for each DASS diagnostic category. High levels of psychological distress have been reported in 6.8–12.3% of rural populations in previous surveys (43–45). This suggests that the higher than normal levels reported in our study are normal for the population sampled, that is, higher for the Australian population but normal for the rural population.

This study reported more than half of the participants were not satisfied with the market accessibility for non-replacement male calves predominately due to poor economical outcomes. Less profitable farming enterprises have previously been associated with negative wellbeing and psychological distress among producers (46). It is possible that the economic burden of non-replacement male calves may have an effect on some psychological parameters among owners and managers of dairy enterprises; however, within this questionnaire, the participants were not asked to report on overall enterprise profitability.

Breed and market were observed to have an influence on profitability in the BN analysis. Figure 4 depicts the probabilities reported from the BN model in relation to the profit achieved by different breeds of non-replacement male calves. All breeds had the ability to be very profitable if certain market requirements were sought, even among Jersey cattle. In this study, many participants were maximizing profitability of non-replacement calves by using a beef sire. Using a beef sire has potential to achieve carcass weight specifications faster, reducing input costs and achieving higher profitability (47).

All markets (Figure 5) in this study had some participants report they were very profitable. Although some markets were likely to be reported as more profitable than others. This suggests there is a possibility for all markets to achieve profitability. The most profitable market reported for non-replacement male calves was pasture finish, on the property of origin. This result aligns with consumer preferences. It avoids euthanasia and selling calves to early-life markets (48, 49). However, dairy-bred animals are known to have higher maintenance energy requirements for growth (50). Pasture-finish dairy cattle could take a longer period of time to reach market requirements, effecting short-term profitability (50). Although, long-term profitability could be achieved as there is greater uniformity among dairy beef carcasses in relation to weight and fat distribution than traditional beef carcasses (51). In Australia, all carcasses are graded against a set of standardized criteria developed by AUS-MEAT (52) to determine the quality of produce generated from both pasture-fed and grain-fed beef systems. Uniformity achieved among dairy-bred carcasses could be advantageous for market requirements and generate more profit.

The participants in this study emphasized their concerns regarding the suitability of the Australian beef grading system for dairy-bred animals. There is a stigma that dairy beef is discounted by processers worldwide and inferior to traditional breeds of beef cattle (3, 50, 53). Dairy steers have been reported to have different dressing percentages, carcass yields, muscling, and intramuscular fat (IMF) distribution due to early maturation in comparison to beef breeds. This may not be accounted for in grading systems and therefore undervaluing carcasses (50, 53, 54). Despite this, previous studies suggest the lean meat yield and meat quality of dairy beef produced under the same conditions can achieve similar carcass outcomes and produce premium quality carcass outcomes due to high amounts of IMF (53–55). A different classification system for dairy beef carcasses may need to be introduced in abattoirs to suit dairy-bred animals (50).

The participants reported they wanted to satisfy both the industry standards and Australian consumers in relation to management strategies related to non-replacement male calves. They also agreed dairy beef products can achieve both premium quality and welfare-friendly outcomes but are unsure whether there is specific demand surrounding dairy beef products. There are shared values between the participants in this study and Australian beef consumers, both agreeing high animal welfare standards are of importance (56).

## Conclusion

This study has assessed the complex interrelationships of practices and attitudes surrounding non-replacement male calf management inclusive of euthanasia practices and the perceived impacts on producer wellbeing. Euthanasia was not identified as common practice among the Australian dairy industry, but when practiced, there may be associated negative effects on wellbeing and psychological parameters of personnel involved. As a cohort, dairy producers reported higher levels of psychological distress than the Australian general population, although this may be normal for rural living. A majority of dairy producers are still not satisfied with market access for non-replacement male calves due to poor economical outcomes however have the potential to maximize profitability through beef sires. All non-replacement male calf breeds and markets accessed were reported by some participants to be profitable, indicating there is potential to maximize the economic benefits among the dairy beef supply chain. However, there is still stigma that dairy beef gets discounted at slaughter. The participants supported this concern questioning the suitability of the Australian beef grading system for dairy-bred animals. Overall, dairy producers want to satisfy both the industry standards and Australian consumers who share similar values, as well as achieve premium and welfare-friendly beef product outcomes for non-replacement male calves.

## Data availability statement

The original contributions presented in the study are included in the article/Supplementary material, further inquiries can be directed to the corresponding author.

## Ethics statement

The studies involving human participants were reviewed and approved by Charles Sturt University Human Research Ethics Committee (Protocol H20352). Written informed consent for participation was not required for this study in accordance with the national legislation and the institutional requirements.

## Author contributions

VV, AS, MC, and JQ collaboratively designed the study. VV collaborated with Spatial Data Analysis Network (SPAN), Charles Sturt University, Wagga Wagga, and GX at the Quantitative Consulting Unit (QCU), Charles Sturt University, Wagga Wagga, to collect and analyze the data. VV wrote the first draft of the manuscript. All authors contributed to the review of the manuscript and approve the submitted version.

## Funding

VV was supported by an Australian Research Training Program Scholarship from Charles Sturt University. MC and JQ are supported by funding from Meat & Livestock Australia.

## Conflict of interest

The authors declare that the research was conducted in the absence of any commercial or financial relationships that could be construed as a potential conflict of interest.

## Publisher's note

All claims expressed in this article are solely those of the authors and do not necessarily represent those of their affiliated organizations, or those of the publisher, the editors and the reviewers. Any product that may be evaluated in this article, or claim that may be made by its manufacturer, is not guaranteed or endorsed by the publisher.
