# Supplementary material for: Producer practices and attitudes: Non-replacement male calf management in the Australian dairy industry
Source: Front Vet Sci. 2022 Sep 20;9:979035. doi: 10.3389/fvets.2022.979035 (PMC9530997; doi:10.3389/fvets.2022.979035)
Supplement: Supplementary file 1 [file Data_Sheet_1.docx]

Supplementary Material

# **Supplementary Tables**

## **Supplementary Tables**

**Supplementary Table 1.** Variables included in the Bayesian network (BN) model (Figure 2) to investigate the relationships between knowledge, practice and attitudes of Australian dairy producers surrounding non-replacement male calves as well as non-replacement male calf supply chain profitability in relation to breed and market type.

| Variable name | Variable meaning | Levels in BN model | Level description |
| --- | --- | --- | --- |
| *Demographics Variables* | | | |
| ID | Random allocated participant ID | ID1 upto ID127 | ID1 up to ID127 |
| Gender | Gender of participant | Male  Female | Male  Female |
| Age | Age of participant in years | 18to29 30to39 40to49 50to59 60to69 70to79 | 18-29 30-39 40-49 50-59 60-69 70-79 |
| Education | Highest level of education attained | < yr10  Yr10  Yr12  Cert_or_dip  Bach_deg  Postgrad  Other | Below year 10 or equivalent  Year 10 or equivalent  Year 12 or equivalent  TAFE Certificate or diploma  Bachelor’s degree  Postgraduate Degree  Other |
| Origin_birth | Participants’ origin of birth | AU  OCE  UK  EU  N America  Other | Australia  Oceania (inclusive of New Zealand)  United Kingdom  Europe  North America  Other |
| Yr_Au_Res | Participants’ number of years of residence in Australia if from “other” origin | 10_14  15_19  20plus  AU | 10-14  15-19  20 or more  Australian origin of birth |
| Owner_manager | Participants’ status of employment | Owner  Manager  Both | Owner  Manger  Both owner and manager |
| Yr_in_industry | Number of years participant has been involved in dairy industry | 1_5  6_10  11_20  21_30  30plus | 1-5  6-10  11-20  21-30  30 or more |
| Yr_in_enterprise | Number of years participant has been an owner and/or manager of current enterprise | <1  1_5  6_10  11_20  21_30  30plus | Less than 1 year  1-5  6-10  11-20  21-30  30 or more |
| Location | Location of dairy enterprise | NSW  VIC  QLD  TAS SA  WA | New South Wales  Victoria  Queensland  Tasmania  South Australia  Western Australia |
| Number_cows | Number of cows in milking herd | 1_99  100_499  500_1000  1000+ | 1-99  100-499  500-1000  1000 or more |
| Breed | Types of breeds in milking herd | HO  JE  AR  Other | Holstein  Jersey  Aussie Red  Other dairy breeds including crossbreds |
| Breed_percentage | Percentage of each breed in milking herd | 10 to 20  20  20 to 60  60 to 80  80 to 100  100 | 10% to 20%  20%  20% to 60%  60% to 80%  80% to 100%  100% |
| *Practice Variables* | | | |
| *NB. Not applicable - Respondent did not answer question due to skip logic* | | | |
| Euth_curr | Dairy enterprises current euthanasia practices of non-replacement male calves on-farm | None  Some  All | Non-replacement male calves are NOT euthanised on-farm  SOME non-replacement male calves are euthanised on-farm  ALL non-replacement male calves are euthanised on-farm |
| Euth_pre | For enterprises that currently do not euthanise; was it a previous practice | Yes  No | Yes, euthanasia has previously been a practice on-farm  No, euthanasia has never been a practice on-farm |
| Euth_pre_rsn | Reason for previously euthanising | Market_value  Market_access  Drought  Facilities_resources  Calf_Size | No market value/ demand for calves  No saleable markets to access for calves  Drought condition effects on resource availability  Lack of facilities and resources to rear calves  Size of calf too small for sale |
| Euth_reintro | For enterprises that currently do not euthanise; will euthanasia be reintroduced | Yes  No  Not_sure | Yes, euthanasia will be reintroduced as a practice on-farm  No, euthanasia will not be reintroduced as a practice on-farm  Unsure is euthanasia will be reintroduced as a practice on-farm |
| Euth_reintro_rsn | Reason to reintroduce euthanasia if required | Market_value  Market_access  Drought  Facilities_resources  Calf_size | No market value/ demand for calves  No saleable markets to access for calves  Drought condition effects on resource availability  Lack of facilities and resources to rear calves  Size of calf too small for sale |
| Euth_cease | For enterprises that currently euthanise some or all calves; is there intention for euthanasia to cease | Yes  No | Yes, there is intention for euthanasia of calves to cease in the future  No, there is not an intention for euthanasia of calves to cease in the future |
| Euth_cease_rsn | Intended reason to cease euthanasia | Market_value  Facilities_resources  Beef_X  Sexed_seamen  Personal_intention | Increase in market value/demand of calves  Increase facility and resources to rear calves  Introduce more beef cross bred animals to target more valuable markets  Greater use of sexed seamen  Personal intention to cease euthanasia |
| Euth_cont_rsn | Intended reason to continue euthanasia | Market_value  Facilities_resources  Calf_size  Small_quantity  Calf_stress  Calf_ Health | No market value/ demand for calves  Lack of facilities and resources to rear calves  Size of calf too small for sale  Not enough calves to batch together to sell  Less stress on calf to euthanise on-farm than send on truck at 7 days old  Poor health related euthanasia |
| Euth_personnel | Personnel in dairy enterprise that currently or previously performed euthanasia of non-replacement male calves on-farm | Respondent  Other_employees  Both  Sent_away | Respondent performed all euthanasia on-farm  Other employees perform all euthanasia on-farm  Both respondent and other employees perform euthanasia on-farm  Calves sent away from property for euthanasia (bobby truck) |
| *Attitudes variables (Euthanasia related)* | | | |
| Euth_resp_  wellbeing | Participant’s ______ euthanasia has a negative effect on their own wellbeing and/or mental health | 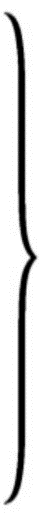  Strongly_agree  Agree Neither_agree_ disagree  Disagree  Strongly_ disagree | Strongly agrees  Agrees  Neither agrees nor disagrees  Disagrees  Strongly disagrees |
| Euth_other_ wellbeing | Participants’ perception ______ euthanasia has a negative effect on employees’ wellbeing and/or mental health |  |  |
| *Attitudes variables continued (Management practice related)* | | | |
| Euth_humane | The dairy's euthanasia practices of non-replacement male calves are humane. | 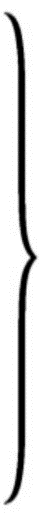  Strongly_agree  Agree Neither_agree_ disagree  Disagree  Strongly_ disagree | Strongly agree  Agree  Neither agree nor disagree  Disagree  Strongly disagree |
| NRMC_  management | I am satisfied with the dairy’s management practices of non-replacement male calves. |  |  |
| NRMC_welfare | The welfare of the dairy's non-replacement male calves is important to me. |  |  |
| Management_  satisfation | I find satisfaction in good management of non-replacement male calves. |  |  |
| NRMC_markets | I am satisfied with the markets the dairy can access for non-replacement male calves. |  |  |
| Industry_standards | The dairy's management practices of non-replacement male calves satisfies industry standards. | 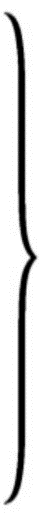  Strongly_agree  Agree Neither_agree_ disagree  Disagree  Strongly_ disagree  Unsure | Strongly agree  Agree  Neither agree nor disagree  Disagree  Strongly disagree  Unsure |
| Australian_  consumer | I want the dairy's management practices of non-replacement male calves to satisfy the Australian consumer. |  |  |
| *Attitudes variables continued (Dairy beef related)* | | | |
| DB_premium | Dairy-bred animals can produce premium beef products. | 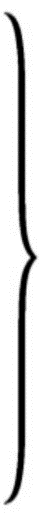  Strongly_agree  Agree Neither_agree_ disagree  Disagree  Strongly_ disagree  Unsure | Strongly agree  Agree  Neither agree nor disagree  Disagree  Strongly disagree  Unsure |
| DB_welfare | Beef products from dairy-bred animals are welfare friendly. |  |  |
| DB_demand | There is a high consumer demand for beef products from dairy-bred animals. |  |  |
| DB_consumption | I would consume beef products from dairy-bred animals. |  |  |
| DB_grading_ suitability | The Australian meat grading system is suitable for dairy-bred animals. |  |  |
| DB_Grading_ appropriate | Australian abattoirs grade beef from dairy-bred animals appropriately. |  |  |
| *Market and profitability variables* | | | |
| Market | Market accessed for non-replacement male calves OR if euthanised on-farm | Euthanasia  Bobby_truck  Saleyard_wk_old  Calf_rearer  Vealer_market  Saleyard  Feedlot  Property_grain  Property_pasture | Euthanised on-farm  Sent on bobby truck to slaughter  Sold at sale yard as week old calves  Sold to calf rearers  Sold to vealer market  Sold at saleyard not as week old calf  Sent/sold to feedlot to be finished  Finished on property (grain-based diet)  Finished on property (pasture-based diet) |
| Breed Calf | Breed of non-replacement male calf sent to chosen market | Friesian_holstein  Jersey  Aussie_red  X_bred_dairy  X_bred_beef  Other | Friesian/Holstein  Jersey  Aussie Red  Cross bred (Dairy breeds)  Cross bred (Dairy x Beef breeds)  Other |
| Profitability | Profitability of market and breed combination for non-replacement male calves | Not_profitable  Rarely_profitable  Breakeven  Usually_profitable  Very_profitable  Unsure | Not profitable  Rarely profitable  Breakeven  Usually profitable  Very profitable  Unsure if market/breed combination is profitable |
