# Supplementary material for: Producer practices and attitudes: Non-replacement male calf management in the Australian dairy industry
Source: Front Vet Sci. 2022 Sep 20;9:979035. doi: 10.3389/fvets.2022.979035 (PMC9530997; doi:10.3389/fvets.2022.979035)
Supplement: Supplementary file 2 [file Data_Sheet_1.PDF]

Charles Sturt University

Rearing non-replacement male dairy calves in Australia

Invitation

You are invited to participate in a study which aims to understand the knowledge, attitudes and practices of primary producers in regard to bobby calves in Australian dairy systems.

This survey will also assess dairy producer wellbeing and core beliefs surrounding euthanasia of non-replacement male calves.

The study is being conducted by Veronika Vicic, a PhD student, under the supervision of Dr Michael Campbell and Dr Jane Quinn from the School of Animal and Veterinary Sciences, and Dr Anthony Saliba from the School of Psychology at Charles Sturt University.

Before you decide whether or not you wish to participate in this study, it is important for you to understand why the research is being done and what it will involve. Please take the time to read the following information carefully and discuss it with others if you wish.

Researchers:
Ms Veronika Vicic, B. Animal Science (Hons I)
PhD Candidate, Doctor of Philosophy,
School of Animal and Veterinary Sciences,
Faculty of Science,
Charles Sturt University

Dr Michael Campbell
Lecturer in Farming Systems,
School of Animal and Veterinary Sciences,
Faculty of Science,
Charles Sturt University

Prof Anthony Saliba
Professor of Psychology,
School of Psychology,
Faculty of Science,
Charles Sturt University

Assoc. Prof. Jane Quinn
Associate Professor in Veterinary Physiology,
School of Animal and Veterinary Sciences,
Faculty of Science,
Charles Sturt University

Charles Sturt University

Rearing non-replacement male dairy calves in Australia

Participant Information

What is the purpose of this study?
The purpose of this research is to:

1. Enhance the understanding of current farm management practices that would influence the development of a dairy-beef supply chain.
2. Assess dairy producers' current opinions surrounding the market acceptability of beef products from various dairy supply chains.
3. Identify dairy producer wellbeing, ill-being and core beliefs surrounding euthanasia of non-replacement male calves.
4. Use the outcomes of the producer questionnaire to have evidence-based research that will assist overcome issues surrounding bobby calf welfare within the Australian dairy industry.

Why have I been invited to participate?
We are seeking producers currently involved in the dairy industry.

To be eligible you must currently be a producer or manager of a dairy producing farm located in Australia.

Please note that to participate in this research you must be over 18 years old.

Should you choose to participate in this study it is important that you respond to the questionnaire based on your current knowledge, attitudes and practices about your production system. This is so we can accurately gauge what producer's perceptions and production choices are regarding this topic. **Adoption of raising bobby calves is NOT a requirement.**

What does this study involve?
If you agree to participate you should read all the information outlined in this Participant Information section and then proceed to the questionnaire by clicking 'Next'. The topics in this questionnaire include your current knowledge, farm management practices, and producer attitudes towards rearing non-replacement male calves within the dairy industry.

It is expected that the time required to complete the questionnaire should not exceed 15 minutes. The questionnaire is completed anonymously. Specific written consent will not be requested because consent is implied if you submit your responses.

Are there risks and benefits to me in taking part in this study?
There are no anticipated risks to taking part in this study. We do understand that some animal management practices covered in this questionnaire may be a sensitive topic for some producers and assure you that participation in this research is anonymous and voluntary.

A factsheet that summarises the results of this study will be freely available and disseminated by the researcher through various platforms at the conclusion of the research. Platforms will include Facebook, Twitter and ResearchGate.

Participants will have the opportunity to receive a **$20 gift card** that will be distributed to participants following the questionnaire process as a symbol of appreciation for the participant's time and efforts. If you would like to receive the gift voucher, you will be asked to enter your contact details. This information will be separated from your questionnaire responses and will not be linked in anyway. Any contact details provided for this purpose will be destroyed once the gift cards have been sent out.

How is this study being paid for?
The study is being funded by the Australian Government Research Training Program and the Graham Centre for Agricultural Innovation, as part of the PhD student's involvement in this study.

Will taking part in this study cost me anything, and will I be paid?
A **$20 gift card** will be distributed to participants following completion of the questionnaire. It will be funded by the AGRTP scholarship.

What if I don't want to take part in this study?
Participation in this research is entirely your choice. Whether or not you decide to participate, is your decision and will not disadvantage you.

What if I participate and want to withdraw later?
As the questionnaire is anonymous it will not be possible to remove individual responses once submitted.

If you start the questionnaire and wish to withdraw part-way through please close the internet browser and your incomplete survey will not be used in the analyses.

How will my confidentiality be protected?
This survey has been distributed by organisations on the research teams' behalf so as to maintain privacy and confidentiality. The questionnaire is anonymous and it will not be possible to identify you from your answers. Data entered online is protected under SurveyMonkey's privacy policy and security statement (click on the word links for more information)

Any information collected by the researchers will be stored securely and only accessed by the researchers unless you consent otherwise, except as required by law. Information will be stored electronically and be kept secure by password. Access to the data will only be granted to the principal investigator and supervisors. Results from the study will not contain any identifiable information. Strict confidentiality will be maintained. Any information collected will not be passed onto a third party.

Data will be retained for 7 years following final publication at Charles Sturt University, School of Animal and Veterinary Sciences, Wagga Wagga to ensure adequate time for analysis and completion of the investigation.

Contact details collected for dissemination of gift cards will be kept separately, and will not be linked to your questionnaire responses. Any contact details provided for this purpose will be destroyed once gift cards have been disseminated.

What will happen to the information that I give?
Information collected will be collated and analysed to investigate the aims outlined above. Findings from this project may be disseminated via industry media releases and will be submitted to scientific journals for publication and may also be presented at conferences either as an oral or poster presentation. Outcomes from the project will also form a chapter for Veronika Vicic's PhD thesis. Individuals will not be identifiable in any material disseminated during or following this research.

The information will be stored for 7 years following final publication. This provides adequate time for the research to be conducted and re-evaluated if required and for comparison studies over time to be conducted if necessary.

What should I do if I want to discuss this study further before I decide?
If you would like further information, please contact the principal researcher:

Ms Veronika Vicic
Tel: +61412431336
Email: vvicic@csu.edu.au

Who should I contact if I have concerns about the conduct of this study?

Charles Sturt University's Human Research Ethics Committee has approved this project [Protocol number: H20352]. If you have any complaints or reservations about the ethical conduct of this project, you may contact the Committee through the Ethics and Compliance Unit via the following contact details:

The Governance Officer
Human Research Ethics Committee
Ethics and Compliance Unit
Locked Bag 588
Wagga Wagga NSW 2678
Tel: (02) 6933 4213
Email: ethics@csu.edu.au

Any issues you raise will be treated in confidence and investigated fully and you will be informed of the outcome.

What do I need to do next?

Please read the following consent statements

- I agree to participate in the above research project and give my consent freely;
- I am over the age of 18 years;
- I consent to participating in this survey;
- I understand that the project will be conducted as described in the Participant Information above;
- I understand that my responses will be saved and submitted as the questionnaire progresses, and they are unable to be withdrawn;
- I understand that my personal information will remain anonymous to researchers and will only be used to send the gift card and will then be destroyed;
- I have had the opportunity to have questions answered to my satisfaction.

If you are happy to participate in the survey, please click on the "Next" button located at the bottom of this section. This will take you to the start of the questions. Once you start the survey it should be complete in one session as you will not be able to close it and resume at a later time.

If required, you can close the survey at any stage, simply close the page or browser window.

Thank you for considering this invitation.

Please print a copy of this information to keep for your records.

Charles Sturt University

Rearing non-replacement male dairy calves in Australia

Support Services

It is a priority of the researchers to ensure the wellbeing of participants is placed above the goals of the research project.

The research team understands there is a negligible risk associated with partaking in this study and that some animal management practices covered in this interview may be a sensitive topic for some producers and may evoke feelings of distress or unease.

Support Services:

Rural and Remote Mental Health
Tel: 1300 515 951
Website: https://www.rrmh.com.au/

Rural Adversity Mental Health Program (RAMHP)
Tel: (02) 6363 8444
Website: http://www.ramhp.com.au/

Lifeline
Tel: 13 11 14
Website: https://www.lifeline.org.au

Beyond Blue
Tel: 1300 224 636
Website: https://www.beyondblue.org.au/

Headspace
Website: https://headspace.org.au

Black Dog Institute
Website: https://www.blackdoginstitute.org.au/

Charles Sturt University

Rearing non-replacement male dairy calves in Australia

Screening

* 1. Do you currently own and/or manage a dairy property?

- ○ Owner
- ○ Manager
- ○ Both own and manage
- ○ No

Charles Sturt University

Rearing non-replacement male dairy calves in Australia

SECTION 1A: Demographics - Personal

* 2. What is your gender?

- Male
- Female
- Other

* 3. What Is your current marital status?

- Single
- In a relationship
- De Facto
- Married
- Separated
- Divorced
- Widowed

* 4. In which state or territory is your permanent residence?

- NSW
- VIC
- QLD
- SA
- WA
- NT
- ACT
- TAS

* 5. What is your residential postcode?

(Note: The location of your residence will not be published in any findings. It will only be used as an indication of where participants are, in terms of population sizes.)

* 6. What is the highest level of education you have attained?

- Below Year 10 or equivalent
- Junior high school - Year 10 or equivalent
- Senior high school - Year 12 or equivalent
- TAFE Certificate or Diploma
- Bachelor’s degree
- Postgraduate studies
- Other (please specify)

* 7. In what year were you born?

* 8. In which region where you born?

- Australia
- Oceania, inclusive of New Zealand
- UK
- Europe
- Asia
- North America
- South America
- Africa

SECTION 1A (cont.)

* 9. In what year did you arrive in Australia to permanently live here?

Charles Sturt University

Rearing non-replacement male dairy calves in Australia

SECTION 1B: Demographics – Work-related

* 10. How long have you worked in the dairy industry?

- One year or less
- 1-5 years
- 6-10 years
- 11-20 years
- 21 - 30 years
- More than 30 years

* 11. How long have you owned and/or managed the dairy where you currently work?

- One year or less
- 1-5 years
- 6-10 years
- 11-20 years
- 21 - 30 years
- More than 30 years

* 12. What is the postcode of the property?

* 13. What proportion of your work (time) is spread across the following roles?
(Please enter whole numbers, without a % symbol, to add to a total of 100)

Milking

Herd health

Reproductive practices

Calf management and rearing

Crop/Grazing management

Other

Charles Sturt University

Rearing non-replacement male dairy calves in Australia

SECTION 1B (cont.)

14. Please describe the 'Other' roles your spend {{ Q13.R6 }}% of your work time on:
(Please skip this question if 0%)

Charles Sturt University

Rearing non-replacement male dairy calves in Australia

SECTION 1B (cont.)

* 15. How many milking cows do you currently own and/or manage on the property?

* 16. What breed/s of cattle, and what proportion (%) of each, make up your dairy herd?

	Percentage of dairy herd:
Friesian / Holstein	
Jersey	
Aussie Red	

Other (for cross breeds and others not listed above, please specify breed/s and % of herd)

Charles Sturt University

Rearing non-replacement male dairy calves in Australia

SECTION 2: Non-replacement male calf practices

This section asks question about practices related to non-replacement calves within your dairy herd.

* 17. Do you currently euthanise non-replacement male dairy calves from the dairy herd?

- ( ) *ALL* non-replacement male calves are euthanised
- ( ) Some non-replacement male calves are euthanised
- ( ) Non-replacement male calves are *NOT* euthanised

Charles Sturt University

SECTION 2: Non-replacement male calf practices

* 18. Do you perform the euthanasia yourself?

- ◯ Yes, I do the euthanasia
- ◯ Both myself and other employee/s do the euthanasia
- ◯ No, other employee/s on the dairy do the euthanasia
- ◯ No, the calves are sent away from the property for euthanasia

Charles Sturt University

SECTION 2 (cont.)

* 19. How do you respond to the following statements:

	Strongly disagree	Disagree	Neither agree nor disagree	Agree	Strongly agree
Performing euthanasia on non-replacement male dairy calves has a negative effect on my overall wellbeing and/or mental health.	○	○	○	○	○
Performing euthanasia on non-replacement male dairy calves has a negative effect on the overall wellbeing and/or mental health of other employee/s.	○	○	○	○	○

Charles Sturt University

SECTION 2 (cont.)

* 20. How do you respond to the following statement:

	Strongly disagree	Disagree	Neither agree nor disagree	Agree	Strongly agree
Performing euthanasia on non-replacement male dairy calves has a negative effect on my overall wellbeing and/or mental health.	◯	◯	◯	◯	◯

Charles Sturt University

Rearing non-replacement male dairy calves in Australia

SECTION 2 (cont.)

* 21. How do you respond to the following statement:

	Strongly disagree	Disagree	Neither agree nor disagree	Agree	Strongly agree
Performing euthanasia on non-replacement male dairy calves has a negative effect on the overall wellbeing and/or mental health of other employee/s.	○	○	○	○	○

Charles Sturt University

Rearing non-replacement male dairy calves in Australia

SECTION 2: Non-replacement calf practices

* 22. Are you likely to cease euthanising non-replacement male dairy calves?

- ( ) Yes
- ( ) No

* 23. Please explain your response:

Charles Sturt University

Rearing non-replacement male dairy calves in Australia

SECTION 2 (cont.)

* 24. What percentage of non-replacement male calves are euthanised and what markets are accessed for the remaining male calves?
(Please select the breed/s and the market/s that are accessed - more than one breed may be selected for each market)

	Market / Euthanised	Breed	Approx. % euthanised or sold to this market	Profitability
1				
2				
3				
4				
5				
6				

Selection of answers for Question 24 and 31 categorized by each column listed below:

Market/ Euthanised:
- Euthanasia
- Bobby Truck
- Saleyard (week old)
- Calf Rearer
- Vealer Market
- Saleyard
- Feedlot
- Property (Pasture Finish)
- Property (Grain Finish)

Breed:
- Holstein/Friesian
- Jersey
- Aussie Red
- X bred dairy
- X bred beef
- Other

Approx. % euthanised or sold to this market:
0-100%

Profitability:
- Very Profitable
- Usually Profitable
- Rarely Profitable
- Break even
- Not Profitable
- Unsure

SECTION 2: Non-replacement male calf practices

* 25. Do you perform the euthanasia yourself?

- ○ Yes, I do the euthanasia
- ○ Both myself and other employee/s do the euthanasia
- ○ No, other employee/s on the dairy do the euthanasia
- ○ No, the calves are sent away from the property for euthanasia

Charles Sturt University

SECTION 2 (cont.)

* 26. How do you respond to the following statements:

	Strongly disagree	Disagree	Neither agree nor disagree	Agree	Strongly agree
Performing euthanasia on non-replacement male dairy calves has a negative effect on my overall wellbeing and/or mental health.	○	○	○	○	○
Performing euthanasia on non-replacement male dairy calves has a negative effect on the overall wellbeing and/or mental health of other employee/s.	○	○	○	○	○

Charles Sturt University

Rearing non-replacement male dairy calves in Australia

SECTION 2 (cont.)

* 27. How do you respond to the following statement:

	Strongly disagree	Disagree	Neither agree nor disagree	Agree	Strongly agree
Performing euthanasia on non-replacement male dairy calves has a negative effect on my overall wellbeing and/or mental health.	○	○	○	○	○

Charles Sturt University

SECTION 2 (cont.)

* 28. How do you respond to the following statement:

	Strongly disagree	Disagree	Neither agree nor disagree	Agree	Strongly agree
Performing euthanasia on non-replacement male dairy calves has a negative effect on the overall wellbeing and/or mental health of other employee/s.	◯	◯	◯	◯	◯

SECTION 2: Non-replacement calf practices

* 29. Are you likely to cease euthanising non-replacement male dairy calves?

- Yes
- No

* 30. Please explain your response:

Charles Sturt University

Rearing non-replacement male dairy calves in Australia

SECTION 2 (cont.)

If you are responding using a mobile phone, please scroll down and across to see all the response options for this question.

* 31. What markets do you currently access for your non-replacement male dairy calves?
(Please select the breed/s and the market/s that are accessed - more than one breed may be selected for each market)

	Market	Breed	Approx. % sold to this market	Profitability
1				
2				
3				
4				
5				
6				

***See selection of answers listed above in Question 24**

* 32. Have you previously euthanised all or some non-replacement male dairy calves?

- Yes
- No

SECTION 2 (cont.)

* 33. Please explain why euthanasia was used:

Charles Sturt University

* 34. Did you perform the euthanasia yourself?

- Yes, I did the euthanasia
- Both myself and other employee/s did the euthanasia
- No, other employee/s on the dairy did the euthanasia
- No, the calves were sent away from the property for euthanasia

Charles Sturt University

SECTION 2 (cont.)

* 35. How do you respond to the following statements:

	Strongly disagree	Disagree	Neither agree nor disagree	Agree	Strongly agree
Performing euthanasia on non-replacement male dairy calves had a negative effect on my overall wellbeing and/or mental health.	◯	◯	◯	◯	◯
Performing euthanasia on non-replacement male dairy calves had a negative effect on the overall wellbeing and/or mental health of other employee/s involved.	◯	◯	◯	◯	◯

SECTION 2 (cont.)

* 36. How do you respond to the following statement:

	Strongly disagree	Disagree	Neither agree nor disagree	Agree	Strongly agree
Performing euthanasia on non-replacement male dairy calves had a negative effect on my overall wellbeing and/or mental health.	○	○	○	○	○

Charles Sturt University

Rearing non-replacement male dairy calves in Australia

SECTION 2 (cont.)

* 37. How do you respond to the following statement:

	Strongly disagree	Disagree	Neither agree nor disagree	Agree	Strongly agree
Performing euthanasia on non-replacement male dairy calves had a negative effect on the overall wellbeing and/or mental health of other employee/s involved.	◯	◯	◯	◯	◯

Charles Sturt University

SECTION 2 (cont.)

* 38. Are you likely to re-introduce euthanasia of non-replacement male dairy calves into your operation again?

- Yes
- No
- Not sure

Rearing non-replacement male dairy calves in Australia

SECTION 2 (cont.)

* 39. Why are you likely to re-introduce euthanasia for non-replacement male dairy calves?

Charles Sturt University

SECTION 2 (cont.)

* 40. In what circumstances would you be likely to re-introduce euthanasia for non-replacement male dairy calves?

Charles Sturt University

SECTION 3: Production practices

The next section is about producer attitudes to production practices.

* 41. How do you respond to the following statement?

	Strongly disagree	Disagree	Neither agree nor disagree / Unsure	Agree	Strongly agree	N/A
The dairy's euthanasia practices of non-replacement male calves are humane.	◯	◯	◯	◯	◯	◯

* 42. How do you respond to the following statements?

	Strongly disagree	Disagree	Neither agree nor disagree	Agree	Strongly agree
I am satisfied with the dairy’s management practices of non-replacement male calves.	◯	◯	◯	◯	◯
The welfare of the dairy's non-replacement male calves is important to me.	◯	◯	◯	◯	◯
I find satisfaction in good management of non-replacement male calves.	◯	◯	◯	◯	◯
I am satisfied with the markets the dairy can access for non-replacement male calves.	◯	◯	◯	◯	◯

* 43. How do you respond to the following statements?

	Strongly disagree	Disagree	Neither agree nor disagree	Agree	Strongly agree	Unsure
The dairy's management practices of non-replacement male calves satisfies industry standards.	◯	◯	◯	◯	◯	◯
I want the dairy's management practices of non-replacement male calves to satisfy the Australian consumer.	◯	◯	◯	◯	◯	◯

Charles Sturt University

Rearing non-replacement male dairy calves in Australia

SECTION 4: Dairy-beef products

* 44. Please indicate how you respond to the following statements:

	Strongly disagree	Disagree	Neither agree nor disagree	Agree	Strongly agree	Unsure
Dairy-bred animals can produce premium beef products.	◯	◯	◯	◯	◯	◯
Beef products from dairy-bred animals are welfare friendly.	◯	◯	◯	◯	◯	◯
There is a high consumer demand for beef products from dairy-bred animals.	◯	◯	◯	◯	◯	◯
I would consume beef products from dairy-bred animals.	◯	◯	◯	◯	◯	◯
The Australian meat grading system is suitable for dairy-bred animals.	◯	◯	◯	◯	◯	◯
Australian abattoirs grade beef from dairy-bred animals appropriately.	◯	◯	◯	◯	◯	◯

Charles Sturt University

SECTION 5: Open questions

45. What words come to mind when you read 'Euthanasia of non-replacement male dairy calves'?

46. What words come to mind when you read 'Premium dairy beef'?

Charles Sturt University

Rearing non-replacement male dairy calves in Australia

SECTION 6

The following sections are to help assess dairy producer wellbeing.

* 47. The following questions ask how satisfied you feel, on a scale from zero to 10.
Zero means you feel completely dissatisfied and 10 means you feel completely satisfied. The middle of the scale is 5, which means you feel neutral, neither satisfied nor dissatisfied.

	0 - Not satisfaction at all	1	2	3	4	5	6	7	8	9	10 - Completely satisfied
Thinking about your own life and personal circumstances, how satisfied are you with your life as a whole?											
How satisfied are you with your standard of living?											
How satisfied are you with your health?											
How satisfied are you with what you are achieving in life?											
How satisfied are you with your personal relationships?											
How satisfied are you with how safe you feel?											
How satisfied are you with feeling part of your community?											
How satisfied are you with your future security?											
How satisfied are you with your spirituality or religion?											

International Wellbeing Group (2006). Personal Wellbeing Index: 4th Edition. Melbourne: Australian Centre on Quality of Life, Deakin University
(http://www.deakin.edu.au/research/acqol/instruments/wellbeing_index.htm)

Charles Sturt University

Rearing non-replacement male dairy calves in Australia

SECTION 7

* 48. Please read each statement and click on a number 0, 1, 2 or 3 which indicates how much the statement applied to you *over the past week*.

The rating scale is:
0 - Did not apply to me at all
1 - Applied to me to some degree, or some of the time
2 - Applied to me to a considerable degree, or a good part of the time
3 - Applied to me very much or most of the time

There are no right or wrong answers. Do not spend too much time on any statement.

	0	1	2	3
I found it hard to wind down	○	○	○	○
I was aware of dryness of my mouth	○	○	○	○
I couldn’t seem to experience any positive feeling at all	○	○	○	○
I experienced breathing difficulty (e.g. excessively rapid breathing, breathlessness in the absence of physical exertion)	○	○	○	○
I found it difficult to work up the initiative to do things	○	○	○	○
I tended to over-react to situations	○	○	○	○
I experienced trembling (e.g. in the hands)	○	○	○	○
I felt that I was using a lot of nervous energy	○	○	○	○
I was worried about situations in which I might panic and make a fool of myself	○	○	○	○
I felt that I had nothing to look forward to	○	○	○	○
I found myself getting agitated	○	○	○	○
I found it difficult to relax	○	○	○	○
I felt down-hearted and blue	○	○	○	○
I was intolerant of anything that kept me from getting on with what I was doing	○	○	○	○
I felt I was close to panic	○	○	○	○
I was unable to become enthusiastic about anything	○	○	○	○
I felt I wasn’t worth much as a person	○	○	○	○
I felt that I was rather touchy	○	○	○	○
I was aware of the action of my heart in the absence of physical exertion (e.g. sense of heart rate increase, heart missing a beat)	○	○	○	○
I felt scared without any good reason	○	○	○	○
I felt that life was meaningless	○	○	○	○

*Lovibond, S.H. & Lovibond, P.F. (1995). Manual for the Depression Anxiety & Stress Scales. (2nd Ed.)Sydney: Psychology Foundation.

Charles Sturt University

Final comments and gift voucher

49. Please feel free to provide any further comments on the topic of male dairy calves, or feedback on the survey experience:

* 50. Would you like to receive a $20 gift card for your participation in this survey?
(Note: If you answer 'Yes', your contact details will be requested for delivery of the gift voucher. This information will be separated from your survey responses to protect anonymity.)

- ◯ Yes
- ◯ No

Charles Sturt University

Rearing non-replacement male dairy calves in Australia

Contact details

Note: These details will be separated from your responses to protect your anonymity and confidentiality.

51. Please provide your address so we can mail you the $20 gift card:

Name:

Address:

Charles Sturt University

Rearing non-replacement male dairy calves in Australia

Support Services

It is a priority of the researchers to ensure the wellbeing of participants is placed above the goals of the research project.

The research team understands there is a negligible risk associated with partaking in this study and that some animal management practices covered in this interview may be a sensitive topic for some producers and may evoke feelings of distress or unease.

Support Services:

Rural and Remote Mental Health
Tel: 1300 515 951
Website: https://www.rrmh.com.au/

Rural Adversity Mental Health Program (RAMHP)
Tel: (02) 6363 8444
Website: http://www.ramhp.com.au/

Lifeline
Tel: 13 11 14
Website: https://www.lifeline.org.au

Beyond Blue
Tel: 1300 224 636
Website: https://www.beyondblue.org.au/

Headspace
Website: https://headspace.org.au

Black Dog Institute
Website: https://www.blackdoginstitute.org.au/

Charles Sturt University

Rearing non-replacement male dairy calves in Australia

Thank you

Thank you for taking the time to participate in this survey. Your responses are very valuable and your time is appreciated.

Please click on the '**Done**' button below to finalise your survey.

Thanks again for your participation.
